# Supplementary figures and images for: HTSplotter: An end-to-end data processing, analysis and visualisation tool for chemical and genetic in vitro perturbation screening
Source: PLoS One. 2024 Jan 5;19(1):e0296322. doi: 10.1371/journal.pone.0296322 (PMC10769073; doi:10.1371/journal.pone.0296322)

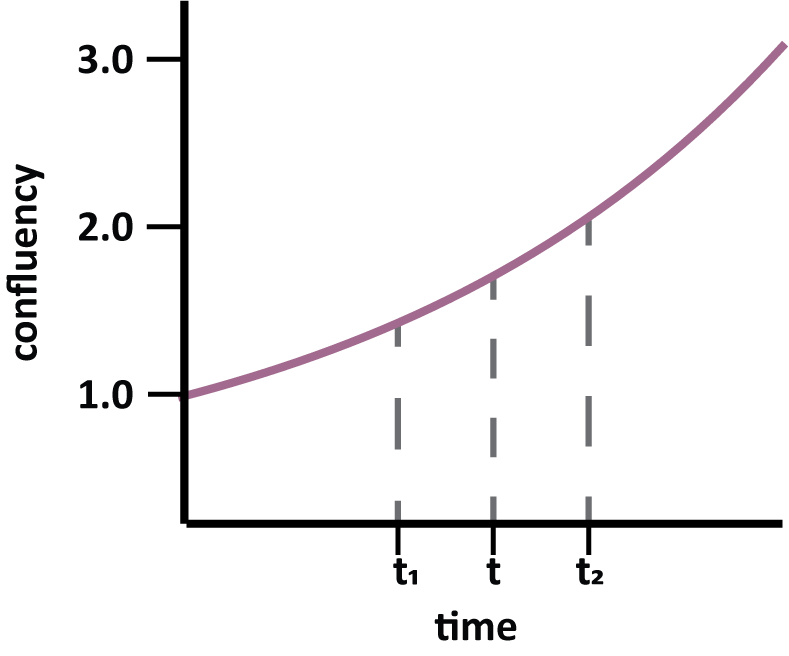

Supplement: S1 Fig — (PNG) [file pone.0296322.s001.png]

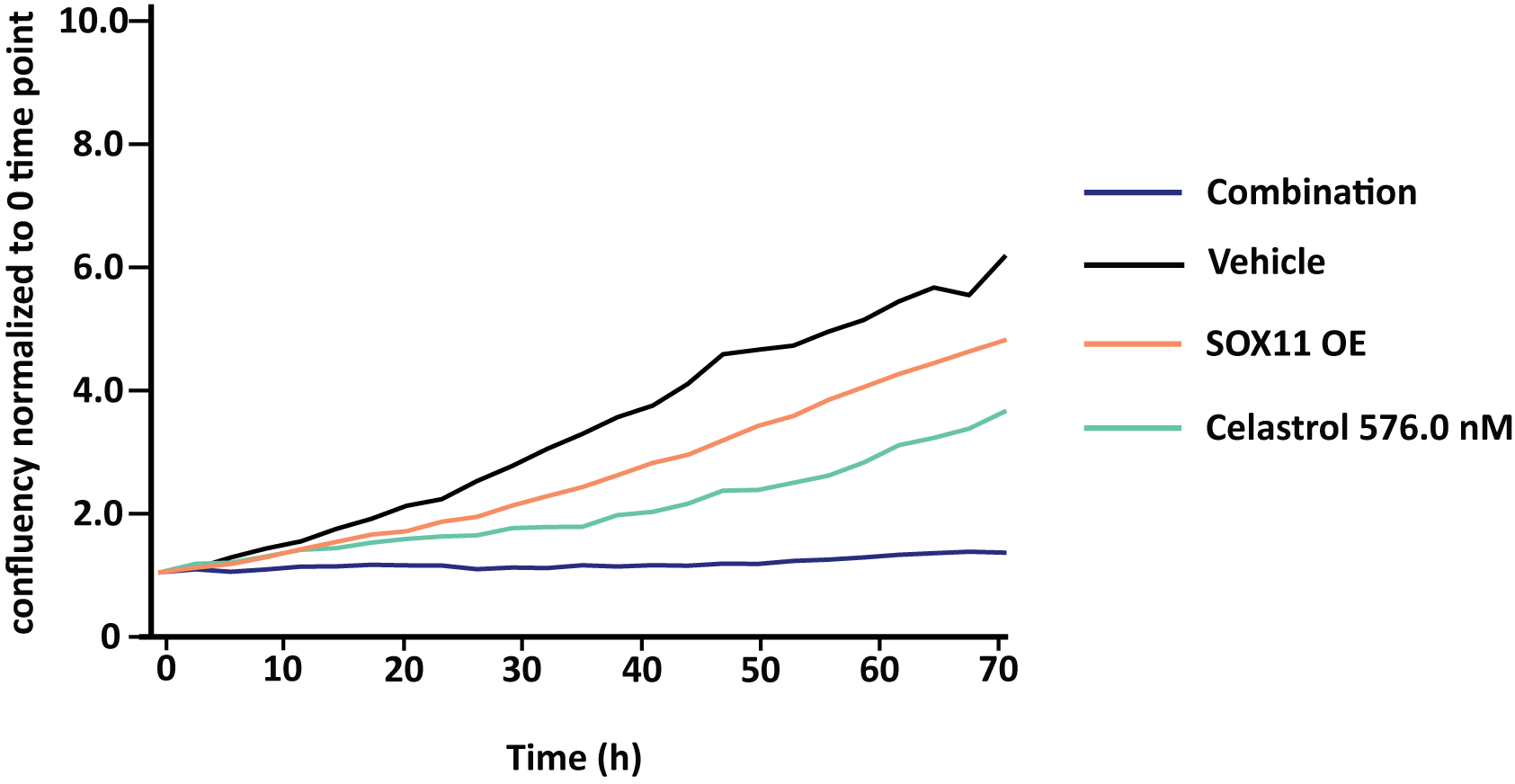

Supplement: S2 Fig — Confluency of each condition normalized to the confluence at the 0 time point. In contrast to the combined condition, each condition alone has an increase confluency over time. (PNG) [file pone.0296322.s002.png]

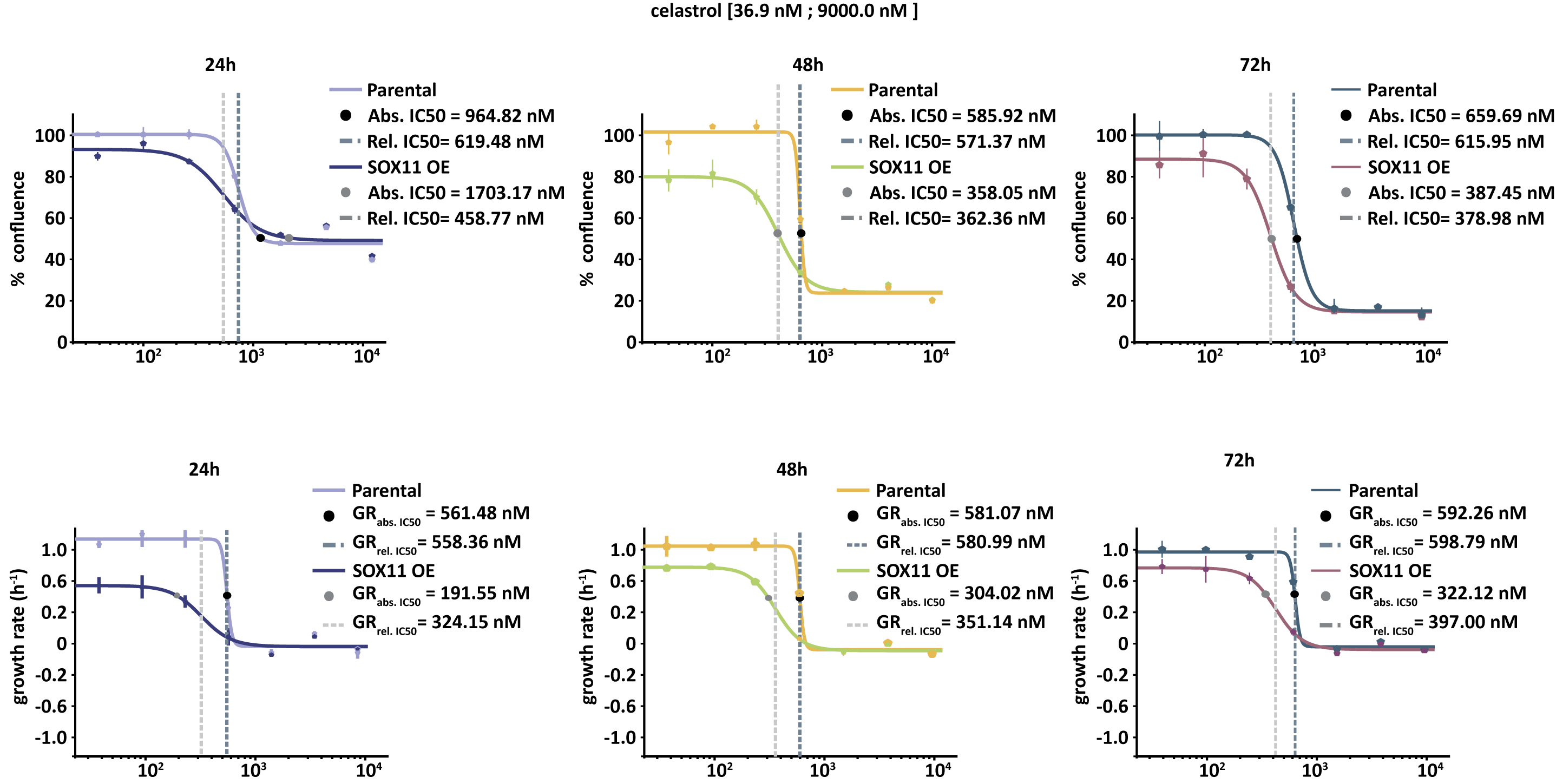

Supplement: S3 Fig — (PNG) [file pone.0296322.s003.png]

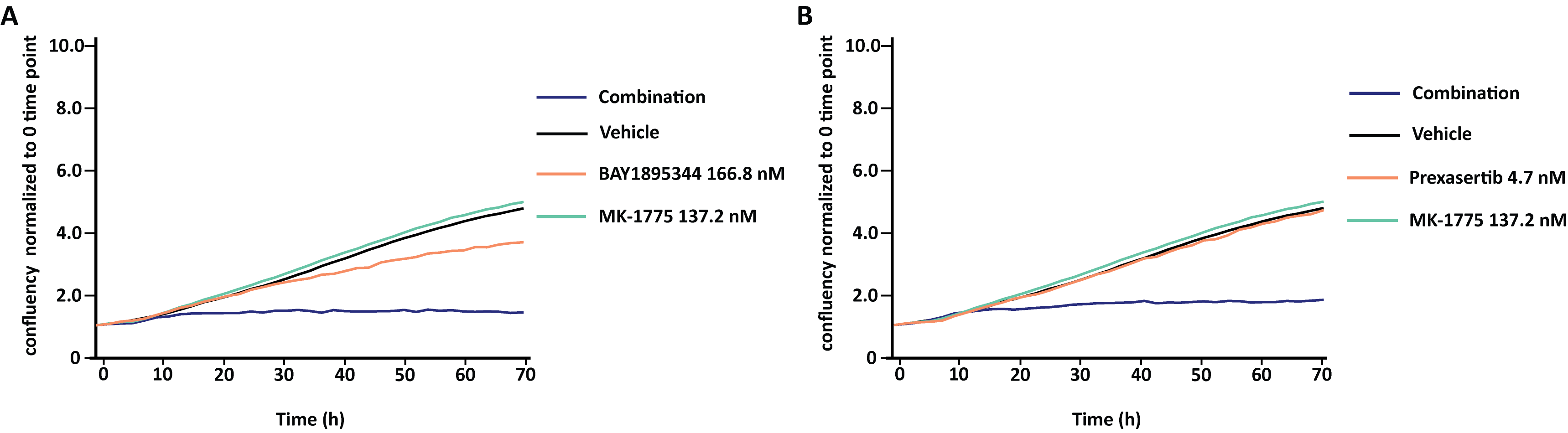

Supplement: S4 Fig — A) An increasing confluency is observed on the BAY1895344 and MK-1775 conditions alone. Over time MK-1775 have a higher confluency, when compared to the control. As the BAY1895344 condition the confluency is lower over time. As for the combined after 10h of treatment the confluency stabilized. B) An increasing confluency is observed for the prexasertib and MK-1775 conditions alone, being the MK-1775 slightly above from the control. The combined condition after 10h of treatment has a constant confluence. (PNG) [file pone.0296322.s004.png]

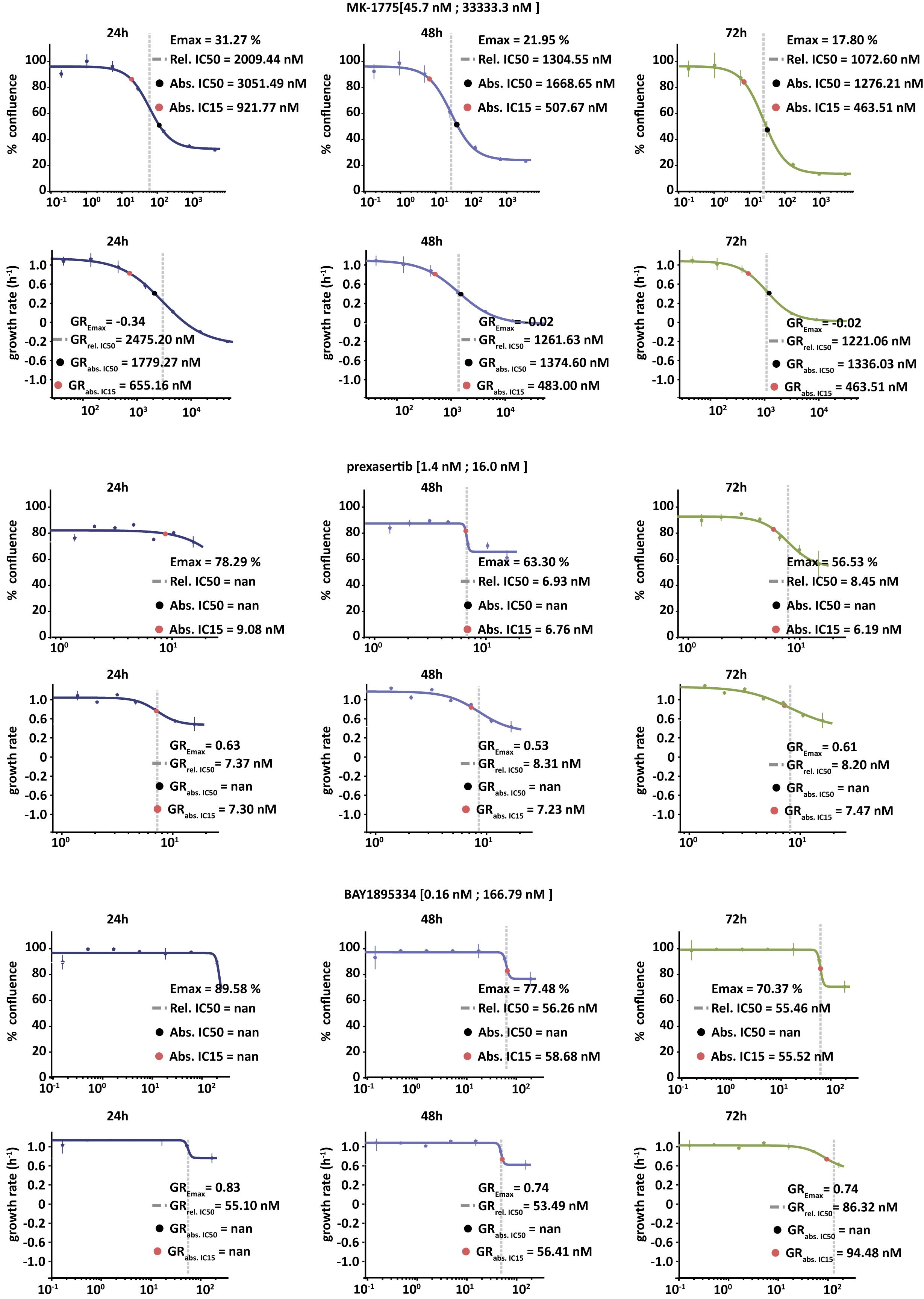

Supplement: S5 Fig — (PNG) [file pone.0296322.s005.png]

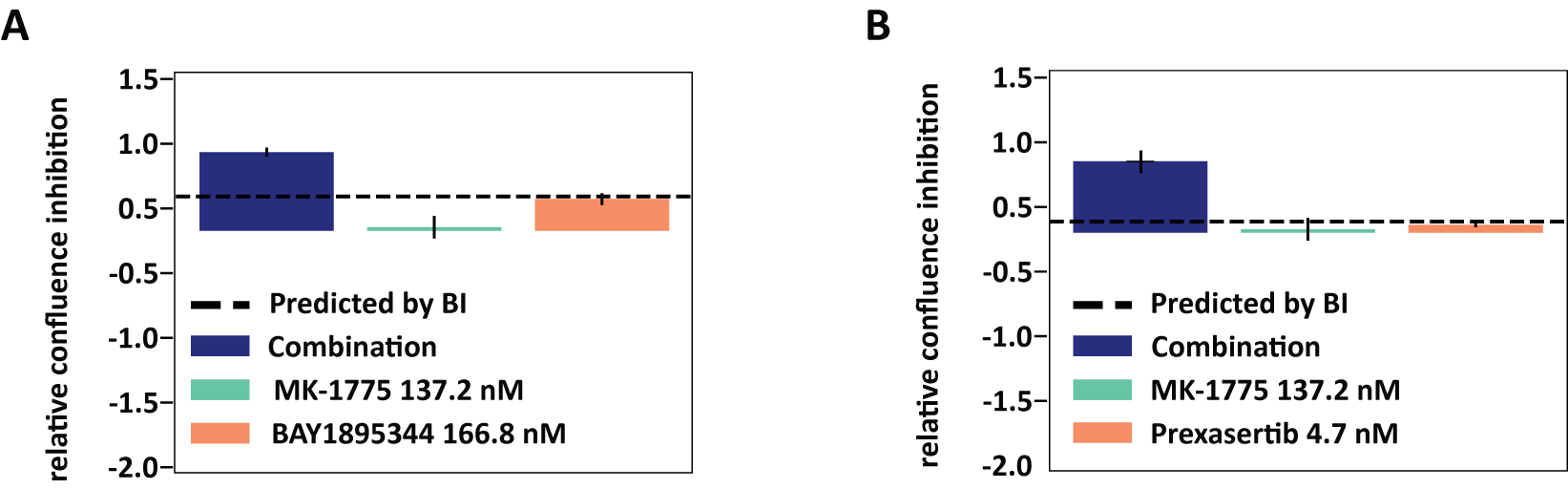

Supplement: S6 Fig — Bar plot, at 72h, of the best dose-effect combination of MK-1775 with BAY1895344 (A) or MK-1775 with Prexasertib (B). The dash line indicates the predicted combination effect, computed according to the BI method, Eq (3). A) Relative confluence inhibition of BAY1895344 at 166.8 nM combined with MK-1775 at 137.2 nM, with a BI score of 0.42. B) Relative confluence inhibition of prexasertib at 4.7 nM combined with MK-1775 at 137.2 nM, with BI a score of 0.56. (PNG) [file pone.0296322.s006.png]

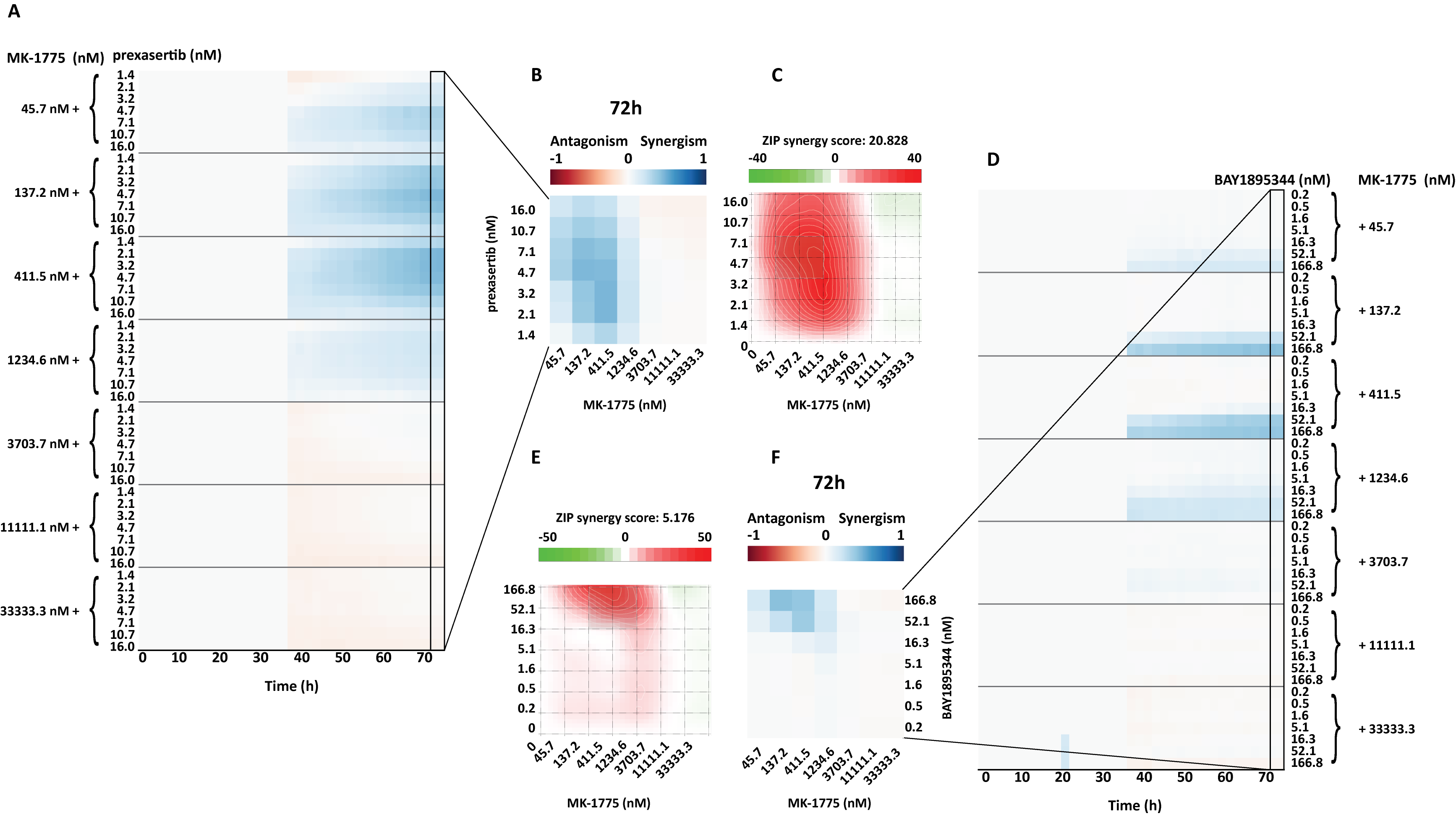

Supplement: S7 Fig — A) HTSplotter heatmap over time and B) at final time point (72h) of dose-effect combination of prexasertib with MK-1775, maximum ZIP score of 0.46 at 72h. C) SynergyFinder Plus heatmap of dose-effect combination of prexasertib with MK-1775, with ZIP score of 17.93 and p value = 4.97x10^-08^. D) HTSplotter heatmap over time and E) at final time point (72h) of dose-effect combination of BAY1895344 with MK-1775, maximum ZIP score of 0.41 at 72h. F) SynergyFinder Plus heatmap of dose-effect combination of BAY1895344 with MK-1775, with ZIP score of 4.92 and p value = 1.26x10^-03^. HTSplotter has a fixed legend scale from -1 to 1. (PNG) [file pone.0296322.s007.png]
